# Subperiosteal Transmission Of Intra-Articular Pressure Between Articulated And Stationary Joints

**DOI:** 10.1038/srep08103

**Published:** 2015-01-29

**Authors:** Mark Pitkin, Raghuveer Muppavarapu, Charles Cassidy, Emil Pitkin

**Affiliations:** 1 Department of Physical Medicine and Rehabilitation, Tufts University School of Medicine, Boston, MA, USA; 2 Depatment of Orthopaedics, Tufts University School of Medicine, Boston, MA, USA; 3 The Wharton School, University of Pennsylvania, Philadelphia, PA, USA

## Abstract

Hydrostatic pressures can be transmitted between synovial capsules. In each of ten rabbits, we simultaneously measured pressure in two joints, one of which was passively ranged, and the other of which was kept stationary. The intra-articular pressure inside the stationary joint changed every time its companion joint was ranged. But the pressure in the stationary joint did not change when the periosteum was transected above the ranged joint. This phenomenon was observed in all four animals that served as their own controls. The study suggests that the intra-articular pressure was transmitted through the space between the periosteum and the bone surface. Alternative explanations, like measurements of venous blood pressure, did not show correlation with hydrostatic pressure changes in the joints. The *Floating Skeleton* concept suggests a biomechanical rationale for this newly observed phenomenon: that there exists a subperiosteal hydrostatic connection of synovial joints, and that this “net” distributes excess pressures among joints through the periosteal sheath to sustain the integrity of the joint contacting surfaces over a lifetime.

It has been documented in the past that if a joint is stationary, the hydrostatic pressure in its synovial capsule remains constant, and if the joint is passively bent or ranged, the pressure changes^1^,^2^,^3^. Articular bending extends part of the joint membrane and changes its curvature; this, coupled with consecutive variations in the effective volume of the capsule, contributes to pressure changes^4^,^5^. The underlying mechanism is described mathematically by Laplace's Law^6^,^7^,^8^.

The purpose of the current study was to determine if the hydrostatic pressure variations in one joint are transmitted along the skeletal system to other joints, including distant joints. Simultaneous measurements in ten rabbits were made of intra-articular pressure in pairs of joints, one of which was put through a range of motion (passive flexion-extension) while the other remained stationary (motionless).

In each animal, we paired joints both ipsilaterally and contralaterally, in the fore and in the hindlimbs. One of the joints was passively put through a cyclic flexion-extension while the other was immobilized and remained stationary (Figure 1; video episode SV1). The experiments confirmed that the pressure in the ranged joints would change, which was in accord with several previous studies^1^,^2^,^3^.

The new result, which to our knowledge has never been reported in the literature, is that pressure changed in the paired stationary joints in response to the ranging of the companion stationary joint, even when the pairs were contralateral.

One explanation for the pressure change in the stationary joint is the possible mechanical action of the muscles and surrounding tissues caused by the cyclic bending in the ranged joint. This mechanical action may affect the shape of the synovial capsule of the stationary joint, and thus explain the change in intra-articular pressure. This conclusion may be correct, however, only for those joint pairs which are close to each other, like the ipsilateral Ankle-Knee, or Knee-Hip pair. This hypothesis cannot explain, however, pressure changes in a stationary distant joint, like the contralateral knee.

Another explanation could be derived from the known phenomenon of correlation between synovial fluid pressure and the pressure in the blood capillary in the synovium^3^,^9^. That mechanism has been observed in single capsules^10^, but can not work for distant joints, due to the morphological route to/from the arterial system. Nevertheless, in our study, simultaneous measurements of venous blood pressure and synovial fluid pressure were conducted; we didn't observe any correlation between the pressure variations in the stationary joint capsules and in the venous system.

The third explanation for the phenomenon observed, and the one we find most compelling, is that the intra-articular pressure was transmitted hydrostatically through the subperiosteal space. To verify, pressures in the stationary knee were measured while the contralateral knee was ranged, both before and after the periosteum was transected 3 cm beyond the groove of Ranvier proximal to the passively ranged knee joint. Before the transection, the passive knee's pressure changed for all four animals taken for that experiment; after the transection, it changed for none of them (video episode SV2). This outcome suggests subperiosteal transmission of pressure changes when the periosteum is intact.

In future studies, the intra-articular pressure network should be more richly mapped. Simultaneous pressure measurements should be made in multiple capsules and along the periosteal sheath for a detailed mapping of pressure transmission from one synovial capsule to another. This network may provide new insight toward understanding the mechanism of intimate contact between the bone heads in a joint. In particular, it may help explain how joint heads safely withstand the surprisingly high reported values of contact pressures, which are close to the limits of cartilage integrity^11^. Other alternative mechanisms, including neurological ones, should be investigated as well. Finally, larger studies are desired, to increase the power to detect statistically significant differences.

## Results

### Intact periosteum

Raw data on pressures measured in the paired joints are presented in Tables 1–4, where pressure changes under ±2 mmHg are indicated in bold. Since the device manufacturer listed ±1 mmHg as the precision of the readings, the bolded observations are those for which no conclusive pressure changes were detected.

**Figure 1:**
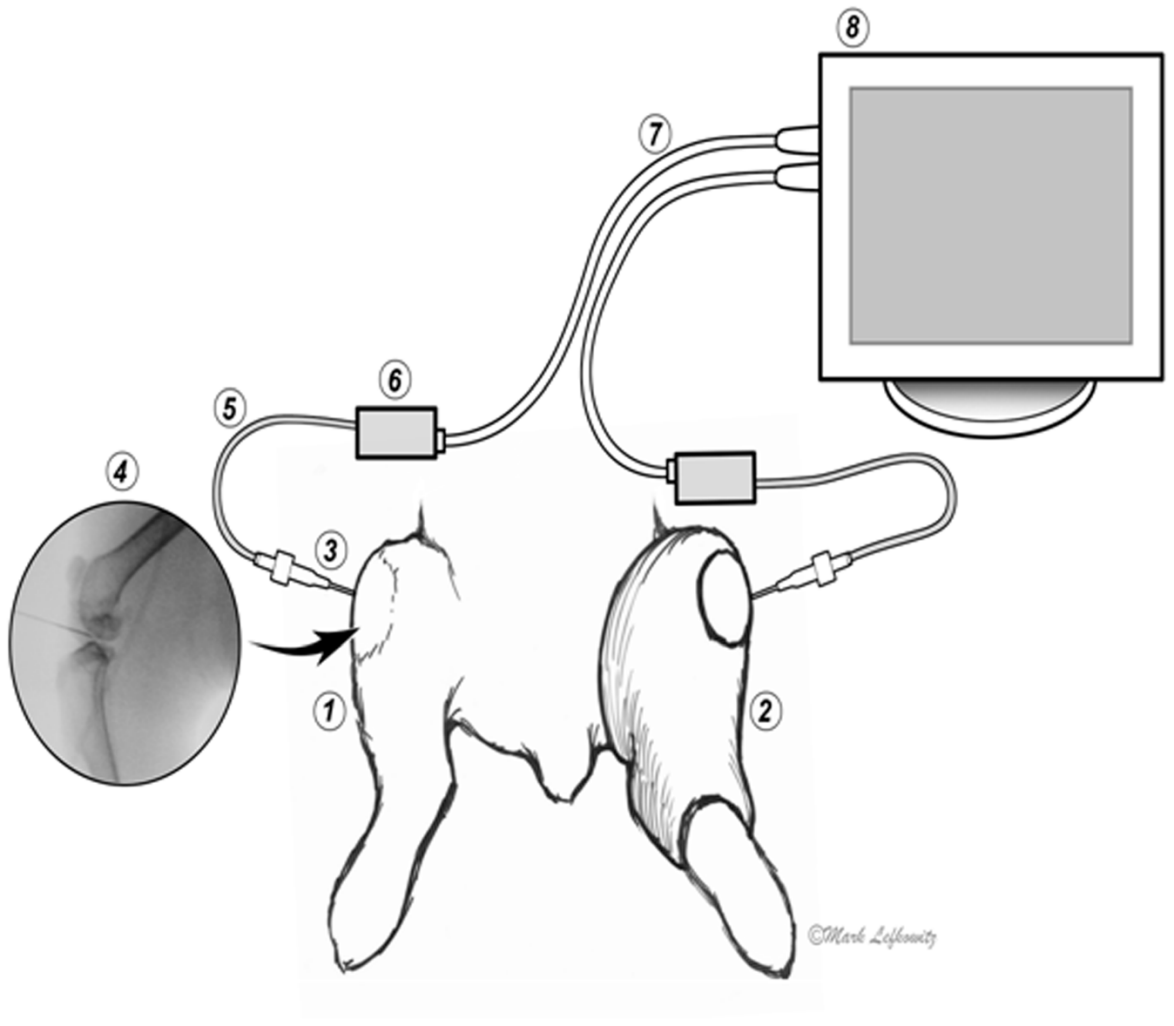
Setup of the experiment for simultaneous pressure measurements in a pair of joints, one of which is movable (1) while the other is immobilized by a cst(2). Cannulas (3) are inserted laterally into the joint capsules under X-ray control (4), and connected via a fluid line (5) to the pressure sensor (6). Signal from the sensor (5) is transmitted via cable (7) to the monitor (8) (copyright -Mark Lefkowitz, Biomedicalvisuals.com).

**Figure 2:**
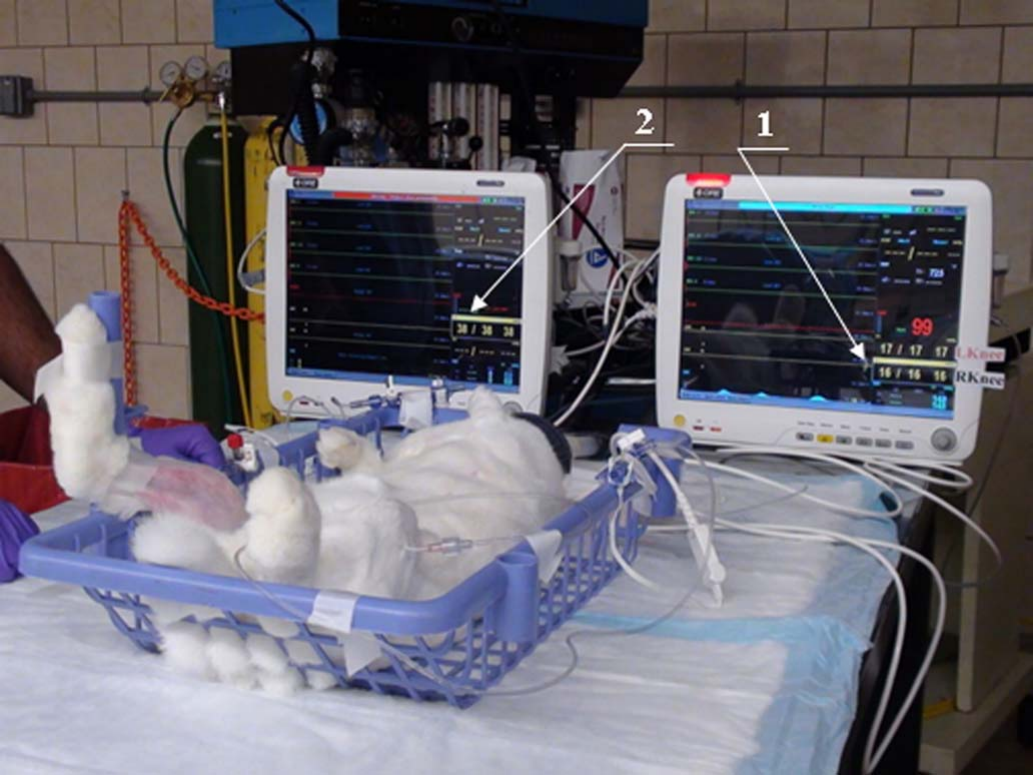
Setup for the measurements of the pressures in the joint capsules. Two lines are connected to the joint capsules via canulae and go to the monitor 1. Venos blood pressure is displayed on the monitor 2. Left hind leg is immobilized with a frame. Right knee is shown to be manually ranged by the investigator.

Pressure conductivity between joints was explored by measuring pressure changes in stationary joints while the joints they were paired with were cyclically bent (Figures 1, 2). The purpose of the initial phase was exploratory, to understand whether articulation in one joint was associated with pressure changes in a distant joint, and to observe these changes in different pairings. Though the first phase was exploratory, care was still taken to more or less balance the number of observations.

Fourteen (14), fifteen (15), fifteen (15), and twenty-two (22), observations were made for the Ankle-Knee, Ankle-Hip, Knee-Elbow and RKnee-LKnee pairings, respectively. In Table 5 are displayed the number and proportion of the trials in which the pressure in the immobilized joint changed while its paired joint was passively ranged. The corresponding percentages for Ankle-Knee, Ankle-Hip, Knee-Elbow, and RKnee-LKnee were 77.2, 85.7, 80, and 86.7, respectively. These numbers are high and surprising. For example, all but two of the measurements in the Ankle-Knee and Ankle-Hip pairings reflected a change in stationary joint pressure.

As a consequence of the exploratory analysis, it was necessary to perform a controlled confirmatory analysis to assess whether the changes in pressure could be explained by natural variation. First, a chi-square test (with three degrees of freedom) revealed that those four proportions did not differ statistically significantly from each other (p = 0.86), so one choice of pairings was not better than another for the confirmatory analysis.

We opted to perform the control study on the contralateral RKnee-LKnee pairing, which circumvented the contribution of the muscles on the shape of the stationary synovial capsule. That contribution could not be ignored in the Ankle-Knee and Ankle-Hip pairs.

### Transected periosteum above the ranged joint

**Figure 3:**
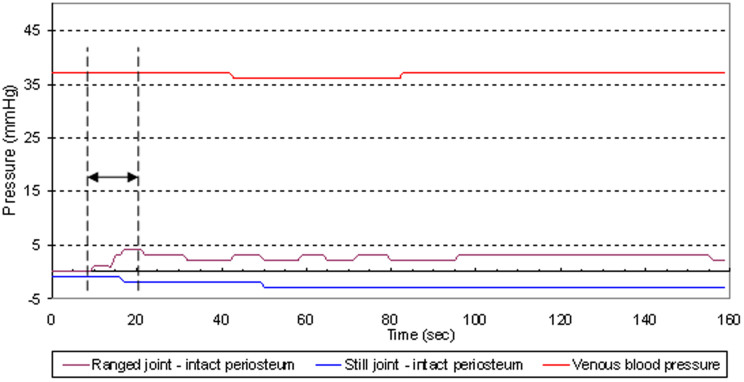
Venous blood pressure and pressure in the ranged and still joints (intact periosteum) in a trial shown in the video file SV1. Double head arrow between two vertical dashed lines indicates a time interval where the driving joint was ranged.

**Figure 4:**
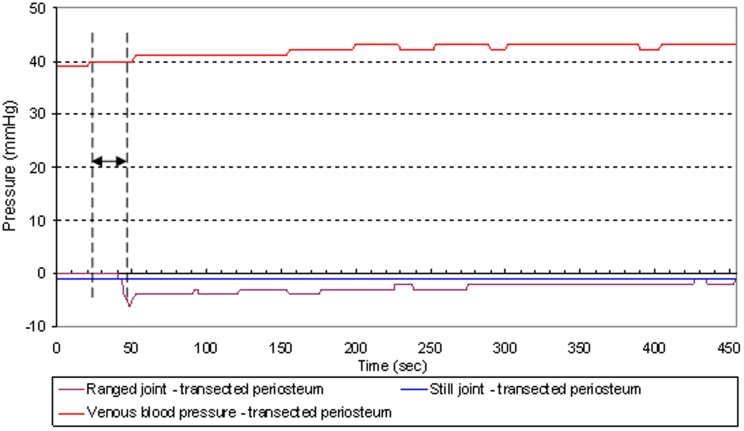
Venous blood pressure and pressure in the ranged and still joints after transecting the periosteum in a trial shown in the video file SV2. Double head arrow between two vertical dashed lines indicates a time interval where the driving joint was ranged.

In the control study, measurements were taken before and after the periosteum was transected distally to the ranged knee joint using the intramuscular approach. For this matched pairs study, the right knee and left knee (RKnee - LKnee) were paired. Along with the intra-articular pressure, we also measured the venous pressure (Figures 3, 4). The purpose was to investigate if the permeability of the synovial wall and fluid exchange between the fenestrated capillary and the synovial capsule^10^ could alternately be the mechanism for pressure transmission between the ranged and stationary joints.

It was found that transecting the periosteum did not affect the pressure variations in the ranged joints (Table 6). However, the pressure changes in the stationary joint capsules were altered dramatically: there were no changes post-transection in the stationary joint capsules in the contralateral knee (Figure 4), although changes were observed pre-transection (Figure 3).

We performed McNemar's test on the RKnee-LKnee observations. McNemar's test is designed for matched pairs, and formally measures whether row and column marginal frequencies are equal^12^. Informally, it is a chi-square test for independence for paired data, and in this case indicates whether the presence of pressure change is different before and after transection.

In all four RKnee-LKnee observations before transection, there were pressure changes in the stationary joint. In all four RKnee-LKnee observations after transection, there were no pressure changes in the stationary joint. The p-value for the test of equal effects was 0.13, *the lowest possible p-value with a sample size of 4*. With a sample size of 4, there was no power to detect a difference — that we found the lowest possible p-value for this design, however, implies the need to consider a similar study with a larger sample size.

Changes in venous pressure during trials were statistically insignificant and did not correlate with pressure changes in the stationary joints (Figures 3, 4).

The fact that pressure in the stationary joints changed in response to the pressure variations in the driving joints, but never changed after the periosteum above the driving joint was transected, suggests that the transmission occurred through the muscle-skeletal system, specifically in the space between the periosteum and the bone surface. The potential role of the blood system in the detected pressure transmission was not supported by our experiments.

## Discussion

The study revealed a phenomenon of variations of the intra-articular pressure in stationary joints in response to pressure changes in passively ranged joints

One possible explanation of the phenomenon is related to the known link between intra-articular pressure and blood pressure in the synovium within single capsules. There are data on the influence of changes in blood pressure in the synovial membrane on the pressure inside that capsule^2^,^13^. Conversely, it has been previously documented that an increase in intra-articular pressure due to effusion can obstruct the flow of blood to the synovium and might be a risk factor for ischemic damage^9^.

Could the vascular system be involved in transmitting pressures between multiple synovial capsules? Several steps would be necessary. First, the pressure changes in one capsule's synovial fluid would be transmitted via the blood plasma to the capillaries located in the capsule's membrane. This first step is quite possible based on the evidence reported above. The next step is difficult to account for, where the pressure gradient is transmitted via the vascular system to distant joints, for example from capsules of the left knee to the right knee. This passage from one capillary bed to the contralateral capillary bed would require passage through the entire circulatory system, which would disperse this gradient. By design, when pressure in the vessels rises or falls, their elastic walls stretch or recoil, changing the vessels' volume. This mechanism is responsible for the dissipation of the pulsation of arterial pressure in the capillaries, and further in the veins, and for the reduction of venous pressure compared with arterial pressure. Thus, even if a vascular contribution exists, it ought to be less significant compared to other routes.

We still experimentally examined whether pressure might be transmitted through the vascular system. Venous pressure didn't show any correlation with the intra-articular pressures in either the stationary or the ranged joints. This discrepancy was likely because the mild correlation effect previous reported was for unhealthy rheumatoid joints and for more extreme conditions compared to our study, and, unlike in our study, certain agents were effused to the capsule^3^,^14^.

Another proposed explanation for the phenomenon observed is the mechanical action from the muscles which impact the position and shape of both joints. While a possible explanation for the closely paired joints like in the Ankle-Knee and Ankle-Hip pairs, the complication of joints co-affected by muscle action does not arise for the more distant pairs of joints, like contralateral knees. It was on these distant joints that we ultimately conducted our control experiments.

In our control trials, the periosteum was transected 3 cm beyond the groove of Ranvier of the ranged knee (so above where the majority of capillaries are located)^15^,^16^. Pressure changes were detected in the stationary joint before the transection, but not after. The mechanical action does not explain this effect for such distant joints, and the transection was above where most of the capillaries are located, so if they were indeed transmitting the pressure gradient they would have continued even after the transection. We concluded that it was the subperiosteal space, not the vascular system, which served as the pathway for pressure transfer.

**Figure 5:**
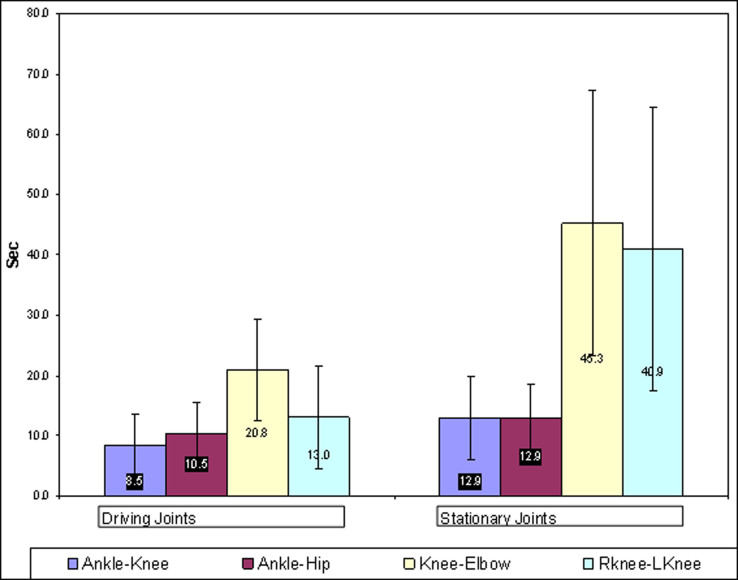
Time delay in pressure change response in the paired driving and stationary joints after the driving joint is first ranged. The bars represent the means and ±1 standard deviation. Left group - driving joints; right group - stationary joints.

The experimental results can be explained by hydrostatic conductivity between joints. Evidence comes in the form of pressure changes (Δp) in the stationary joints after distant joints are cyclically bent (Tables 1–4). According to Pascal's principle, pressure exerted anywhere in a confined incompressible fluid is transmitted equally in all directions without the fluid's flowing. And indeed, synovial or sub-periosteal fluid needn't flow. Detected delays in pressure changes (Figure 5) in the stationary joints may be explained by the non-Newtonian properties of the synovial fluid which is not incompressible and whose viscosity in not constant. In addition, a possible elastic deformation of the synovial capsules and periosteal sheath covering several bones should be considered.

We opted to directly measure pressure in joints instead of relying on radiology, which would require the dispersion of radioactive particles to the areas of interest. Also, radioactive particles are rapidly absorbed by and travel within the blood system.

In our analysis, a pressure transmission was considered to have occurred when a change in the pressure in the stationary joint was no less than ±2 Hg. That amount is in the range reported by other researchers who investigated variations in intra-articular pressure under different conditions. Levick^1^ reported an increase in fluid pressure in the knee over 2 cm H_2_O (1.4 mm Hg) due to acute flexion of the ankle joint in a rabbit. Tarasevicius, et al. reported the mean hydrostatic intracapsular pressure of 2.2 mm Hg in a human hip in 45° of flexion^17^.

The ranging of a joint in our experiment, i.e. its passive flexion-extension, was performed manually, without instrumentation standardizing the angulation and bending moments exerted by the operator. As a result, in three trials we did not observe any pressure change in the ranged joint, attributable to an insufficient amplitude of bending. Data on these trials were excluded from the analysis. An occasional absence of pressure changes in a joint in response to its bending was explained by Wingstrand and colleagues^18^ who found no increase in intracapsular pressure within the normal range of rotation around the axis of the neck of the femur unless the joint was put in positions of extreme rotation.

Our technique of measuring hydrostatic pressure with cannulae inserted into the synovial capsules was similar to methods used by other authors^1^,^4^,^9^,^19^, and we had to address all attendant limitations. For example, there can be leaks where the cannulae are inserted into the membrane that would compromise the joint pressure measurements. To address this concern, we included rest periods between the cycles of flexion-extension articulation without removing the cannulae. During each cycle we recorded pressure changes in both the driving and the stationary joints, and during the rest periods we waited until the pressure returned to the zeroed level. The conclusion was drawn that even if the absolute values of the pressure are affected by the potential leak, the outcomes of the experiment on “yes-or-no” changes in the stationary joint, were not compromised.

A logical question arises about the morphological possibility for hydrostatic conductance between the capsules of distant joints. The periosteum lines the outer surface of all bones except the sesamoid bones and the intra-articular ends of bone^20^,^21^. The subperiosteal fluid can be found in the cambial layer of the periosteum^18^,^22^.

At present very little is known about the response of the periosteum to the mechanical environment or to mechanical stimulation. However, with its modulus of elasticity in various parts in the range of 91.7+/−30.5 MPa and 63.0+/−25.4 MPa^9^,^23^, compared to 0.4 MPa in the venous capillary^24^,^25^, the periosteum can effectively contain the subperiosteal space, which makes it a good candidate for effective transmission of the pressure gradient along the skeleton.

A possible structure for providing and facilitating the hydrostatic connectivity between the synovial joints through the periosteum could be a system of gap junctions. A gap junction consists of intercellular channels providing aqueous continuity between adjacent cells ranging in width from 20 to 40 nm^26^. Since the size of a water molecule is about 0.2 nm, an average gap junction would provide sufficient space for the passage of the synovial fluid from one capsule to another via the periosteum^20^,^27^, while preventing the flow of synovial fluid between capsules.

**Figure 6:**
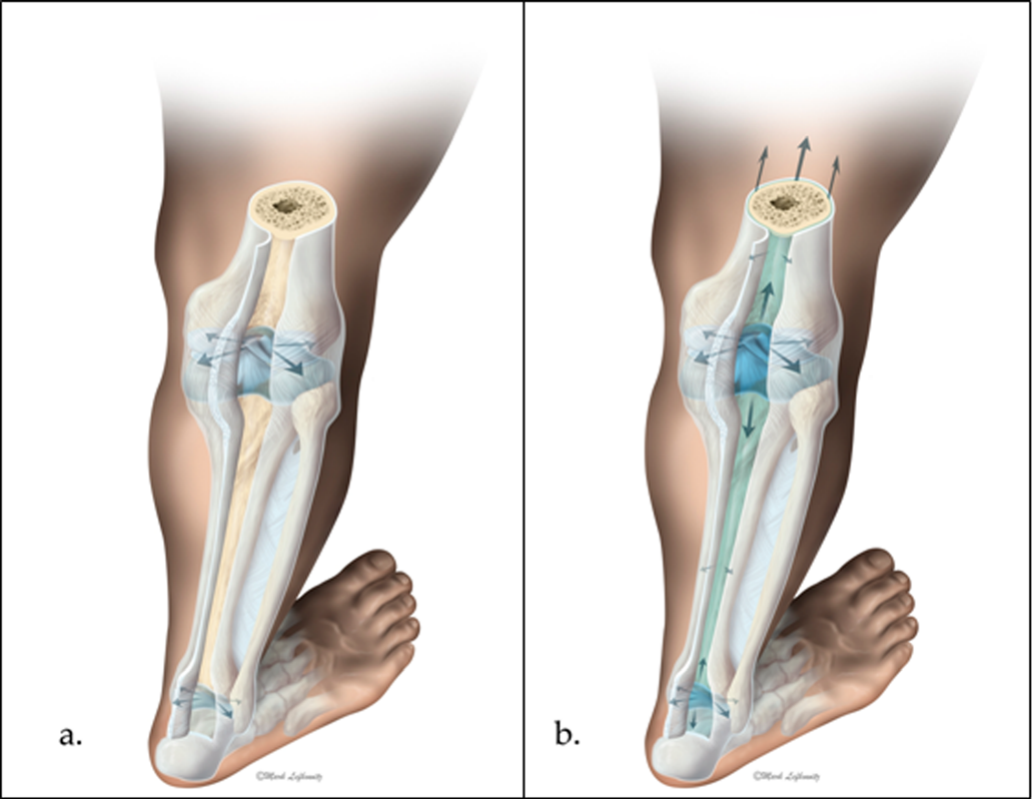
Artistic representation of the subperiosteal hydrostatic system for transmitting and distributing pressure along the skeleton without flow of the synovial and subperiosteal fluid. (a) - Traditional representation of synovial capsules that are hydraulically isolated from each other, where hydraulic pressure (shown with arrows) of the synovial fluidic contents (shown in blue) is applied normally to the corresponding synovial membranes. (b) - Illustration of the Floating Skeleton Concept with the subperiosteal layer of fluid shown in green, as seen through an opening via a longitudinal cut in the periosteum and two synovial membranes. The fluid layer connects the fluidic contents of two synovial capsules and extends beyond them, transmitting pressures between the capsules without the fluid's flow (copyright - Mark Lefkowitz, Biomedicalvisuals.com).

As there is not yet sufficient knowledge about the morphology of the subperiosteal hydrostatic system we are discussing here, the authors commissioned a professional illustrator to create an artistic representation of this system in humans (Figure 6). A subperiosteal layer of fluid is shown in green through the opening after a longitudinal cut in the periosteum and two synovial membranes. That layer connects the fluidic contents (shown in blue) of two synovial capsules and extends beyond them.

While the rabbit animal model used in this study is widely accepted for investigating the human skeleton, the authors realize that primate studies might be more convincing. The latter animal model would be important to better understand the mechanisms that protect joints from overloading and for developing more effective prevention, treatment and rehabilitation modalities.

The demonstrated transmission of pressure traces its origins to the *Floating Skeleton* concept introduced in 1993 by one of the authors^28^, where synovial fluid is not totally contained inside a particular joint capsule as is taught in standard anatomy^29^, but is a part of hydrostatic net which includes synovial capsules and a periosteal sheath covering the bones between them. Such a net can transmit excessive pressure in one joint to the other via the space under the periosteum, without the flow of fluid, by passing through the zone of the groove of Ranvier where the synovium meets the periosteum.

The study presents the first experimental verification of the hypothesis of hydrostatic connectivity of synovial capsules via the periosteum. A new role is therefore suggested for the periosteum, in addition to the previously attributed roles of osteogenesis^23^,^30^ and force transmission from the muscles to the bones^31^,^32^.

The static transmission of pressures along the skeleton could also suggest that the actual contact pressures between the cartilages in a joint are significantly lower than has been estimated with different methodologies. One such methodology is a computerized gait analysis representing the skeleton as a poly-linker with pin joints between solid links^33^. With that approach, and knowing the real-time deformation of the cartilage contact pressure on the talar bone head during one-leg standing^34^ should be up to 4.8 MPa^27^, which is five times greater than the pressure exerted on the ground by a bulldozer or military tank. Gait analysis methodology ignores important characteristics of the morphology of a live joint and simplifies the capsule design to a “dry” pin joint, and measurement of contact pressure in artificial joints lacks the multiple effects of the structure of live cartilage.

Another approach that produces suspicious results involves placing the load or pressure sensors in the contact zone of cadaver specimens or in artificial joint heads in subjects with joint replacement interventions. The pressures in gait on the heads of the instrumented artificial hip joints were found to be in the 5- to 6-MPa range and 18 MPa on average when rising from a chair^35^ reaching the threshold of cartilaginous resistance to the loads in the interval of 14–25 MPa^11^. Such high values of pressure can be explained by the fact that in cadavers, the subperiosteal fluid would have dried out, and artificial joints are not imbued with synovial fluid.

An indication of the role of the walls of an intact synovial capsule in redistributing pressure on the joint cartilages was provided in a recent cadaver study by Jaumard and colleagues^36^. With a new technique which did not require resection of the joint capsule for placing pressure-sensitive films, the facet joint pressure in the cervical spine was found to be half of the previous method. We believe that it is reasonable to expect an even greater reduction in the values of contact pressures in live joints, which can be measured once new noninvasive methods are developed.

## Methods

All experiments were performed in accordance with relevant guidelines and regulations. The protocol for the study was approved by the IACUC of the Pine Acre Rabbitry Farm, Norton, MA, where all experiments were conducted. Ten male New Zealand rabbits weighing 3.5–4 kg were sedated and anesthetized with nitro gas and narcotic intra-venous.

The study was designed to answer two questions: 1) whether the pressure in a stationary joint changes following pressure changes in the articulated joint; 2) if this change is recorded, whether the pressure is transmitted subperiosteally.

To answer the first question we conducted a series of simultaneous pressure measurements in passively ranged and stationary joints. The joints were paired ipsilaterarly: Ankle-Knee, Ankle-Hip, Knee-Elbow, and contralaterally: Right Knee-Left Knee (Fig. 2). The driving (passively ranged) and stationary joints were alternated for each joint pairing, in order to record potential pressure transmission in both directions relative to the articulated joint. To minimize the sedation dose for the rabbits, the duration of experiments for each animal was limited by the time required to conduct measurements in the joint pairs. One trial consisted of 1–3 cycles of 10 flexion-extension articulations with a resting period of 30 seconds between cycles. The flexion-extension articulations were made manually by the surgeon examiner. Joints were put through a full range of motion from full extension to flexion.

The timeline for the pressure changes and the venous blood pressure was recorded. A chart of time delay in pressure change response after the driving joint was first ranged is shown in Figure 5, both for the driving and the stationary joints.

As shown in Fig. 1, one of joints was movable (1) while the other was immobilized with a splint cast (2). Fluoroscopy was used to localize the joint capsules and an 18 gauge A-line catheter (3) was inserted into each of the two joints. The intra-articular pressure reading was obtained via the arterial line (4). The pressure in both capsules was simultaneously measured with two independent pressure transducer systems (5) (Deltran®, Utah Medical Products, Inc., Midvale, UT) with zero drift ≤1.0 mmHg/8 hours after 10 minute warm-up. The output was fed via cable (6) to a DRE Waveline ProVet Multi-Function Patient Monitor (7), DRE Veterinary, Louisville, KY. The baseline pressure was zeroed when both joints were in a neutral position.

Vitals including temperature, blood pressure, heart rate, and oxygen saturation were monitored throughout the procedure. The setup of the experiment for simultaneous measurements of pressure in a pair of joints is further illustrated in Figure 2 and in video episode SV1.

To answer the second question of the study: if the pressure in a stationary joint changes following pressure changes in the articulated joint, whether that pressure is transmitted subperiosteally, we conceived a control experiment with four animals, measuring pressures on contralateral knees in which the periosteum was cut 3 cm above the ranged knee's groove of Ranvier (video episode SV2). We made a small incision at the desired level and chose a spot 3 cm proximal to the knee joint. After the skin incision we identified and divided the iliotibial band (tract) in line with the skin incision. The incision followed the fiber orientation to avoid disruption of function. We dissected between the muscle layers using a lateral approach to avoid vascular structures. The muscle fibers of the *vastus lateralis* are minimal in the distal part of the femur. We incised the muscle fascia, investing the *vastus lateralis* just anterior to the lateral intermuscular septum and elevated the muscle fibers off the septum, working from distal to proximal. This was most easily accomplished by use of a large elevator. We completed an atraumatic elevation of the *vastus lateralis* from the lateral aspect of the distal femur. We then placed retractors to give us visualization of the femoral shaft. We only needed a small window so we could make a circumferential incision in the periosteum. We then closed the fascia of the *vastus lateralis* with a running absorbable suture (vicyrl) and then closed the iliotibial band with a running absorbable suture. Skin was closed with nylon sutures. This is the same approach used in humans for femur fracture plating. However, since we were not plating the rabbit's femur but instead making a small circumferential incision in the periosteum, we were able to minimize the surgical dissection and trauma.

Measurements of the venous blood pressure were conducted to answer the question about the possible influence of the circulatory system of pressure transmission between the joint capsules. The rabbit's ear was chosen as the location for venous pressure measurement due to the large venous plexus present in the ears. Once the area was sterilely prepared and the vein identified, a catheter was introduced into the vein. A transducer was then attached to the catheter via intravenous tubing. Sterile saline was used to flush the line and check that there was a good flow via the tubing into the vein. The transducer was connected to the monitor device, the second DRE Waveline ProVet Multi-Function Patient Monitor, DRE Veterinary, Louisville, KY, as shown in video episodes SV1 and SV2.

## Data analysis technique

Since the sensitivity of the apparatus, according to the manufacturer (Utah Medical Products, Inc., Midvale, UT), was ±1 mmHg, we conservatively counted any reading under ±2 mmHg as 0, or as no change.

The nature of our data informed the statistical methodology employed to analyze it. In our dataset, we have, for each animal and joint pair under consideration, a measurement of the difference in intra-articular pressure inside the passive joint (difference = pressure inside passive joint after other joint was articulated minus pressure inside passive joint before other joint was articulated). Some differences were negative; others were positive; some were equal to zero.

According to the presently accepted theory, the intra-articular pressure in the passive joint should remain unchanged (the before-after difference should be zero) when a distant joint is articulated, since their sacs of synovial fluid are isolated from each other. Therefore, *any* change in pressure – whether positive or negative – we considered as evidence of pressure transmission. A simple mean of pressure change would be an inadequate measure, since changes of, say, (+5 mm, +7 mm, −5 mm, −7 mm) are all interesting and are each indicators of synovial hydrostatic connectivity, but the mean of these 4 potential observations is 0.

With sample size as low as we had, we did not look at the absolute value of the difference in intra-articular pressure, since its distribution would have been non-symmetric, and symmetric and reasonably normal data are required for standard parametric t-tests when sample sizes are small.

We therefore opted for a non-parametric approach to answer the chief question of our study: was there a change in pressure, or wasn't there? Each animal acted as its own control, and so that pressure changes in the same passive joint were measured before and after the periosteum was transected. If there were no mechanism of pressure transmission in the musculoskeletal system, then the presence or absence of pressure change should be unaffected by the transection: if pressure changes before transection, it does change after; and if it does not change before transection, then it should not change after.

The pair of joints for the control study was to be chosen after exploratory analysis. If one pair of joints tended to produce a higher proportion of pressure changes than the other pairs, then we would study that pair in the confirmatory analysis, since a finding of no pressure change post-transection would be more surprising. If the joint pairs did not differ significantly in proportion of passive joint pressure changes, then we would just choose one. We opted for the contralateral Right Knee-Left Knee pairing, to overcome the issue of articular deformation in the passive joint caused by muscle contraction.

To measure the agreement in pressure change before and after transection, we employed McNemar's test for paired data^12^. McNemar's test, heuristically, measures the degree to which responses measured twice (here, before and after periosteal transection) agree with each other. Because we had only four animals available for the control trials, there was no power to detect a difference if it existed. It was determined that we would find the statistical results meaningful only if the p-value was the lowest among all possible p-values for the experiment (0.13) — that is, if 4 pressure changes were observed before transection, and none after. This was in fact the case.

## Supplementary Material

Supplementary Information

Supplementary Information Experiments with intact periosteum

Supplementary Information Experiments with cut periosteum

## Figures and Tables

**Table 1:** Dynamics of pressure change Δp in driving and stationary Ankle-Knee (ipsilateral) pair^1^

Trial #	t_r_	Driving joint	Stationary joint	Δp
1	7	RKnee		10
	10		RAnkle	4
2	6	RKnee		**0**
	10		RAnkle	3
3	9	RAnkle		6
	7		RKnee	4
4	6	RKnee		3
	7		RAnkle	4
5	5	RKnee		6
	21		RAnkle	4
6	4	RAnkle		6
	21		RKnee	21
7	7	RKnee		2
	4		RAnkle	2
8	12	RKnee		3
	18		RAnkle	−3
9	23	LAnkle		−2
	21		LKnee	2
10	3	LKnee		4
	7		LAnkle	−2
11	8	Lknee		3
	20		LAnkle	4
12	9	LAnkle		2
	22		LKnee	6
13	10	LKnee		6
	8		LAnkle	**1**
14	10	LKnee		−3
	6		LAnkle	−6

^1^In Tables 1–5, Δp is a pressure change (mmHg), which occurred in a driving and stationary joint in a response time interval **t_r_** (sec) after beginning of a trial. The **Äp** values below the threshold of ±2 mmHg are indicated in bold.

**Table 2:** Dynamics of pressure change in driving and stationary Ankle-Hip (ipsilatral) pair

Trial#	t_r_	Driving joint	Stationary joint	Δp
1	7	RAnkle		−10
	8		RHip	**−1**
2	10	RAnkle		−9
	11		RHip	**0**
3	19	RHip		−2
	13		RAnkle	4
4	15	RHip		−10
	16		RAnkle	−4
5	4	RHip		6
	3		RAnkle	4
6	13	RHip		6
	7		RAnkle	−2
7	10	RAnkle		6
	17		RHip	6
8	10	RAnkle		16
	20		RHip	9
9	18	RAnkle		5
	23		RHip	5
10	5	RHip		−2
	7		RAnkle	3
11	11	RHip		−12
	17		RAnkle	4
12	5	RHip		6
	9		RAnkle	3
13	7	RHip		−2
	11		RAnkle	3
14	17	RHip		−2
	16		RAnkle	3
15	6	RAnkle		3
	16		RHip	**−1**

**Table 3:** Dynamics of pressure change in driving and stationary Knee-Elbow (ipsilateral) pair

Trial#	t_r_	Driving joint	Stationary joint	Δp
1	24	RKnee		2
	77		RElbow	**−1**
2	22	RElbow		7
	24		RKnee	2
3	15	RKnee		−4
	95		RElbow	−2
4	23	RKnee		−6
	45		RElbow	**0**
5	32	RElbow		6
	45		RKnee	3
6	26	LKnee		6
	40		LElbow	2
7	29	LKnee		4
	50		LElbow	3
8	25	LKnee		8
	35		LElbow	2
9	35	LKnee		6
	45		LElbow	3
10	5	LKnee		10
	15		LElbow	3
11	15	RKnee		−8
	70		RElbow	2
12	20	RKnee		7
	51		RElbow	2
13	14	RElbow		8
	40		RKnee	**1**
14	20	RElbow		2
	31		RKnee	−8
15	7	RKnee		3
	16		RElbow	7

**Table 4:** Dynamics of pressure change in driving and stationary RKnee-LKnee pair

Trial #	t_r_	Driving joint	Stationary joint	Δp
1	29	RKnee		−3
	87		LKnee	−4
2	8	RKnee		3
	15		LKnee	2
3	6	LKnee		9
	27		RKnee	−2
4	15	RKnee		5
	97		LKnee	**1**
5	6	LKnee		3
	10		RKnee	2
6	5	LKnee		22
	25		RKnee	2
7	3	LKnee		−14
	29		RKnee	2
8	4	LKnee		−5
	9		RAnkle	3
9	16	RKnee		−4
	27		LKnee	−3
10	7	LKnee		4
	64		RKnee	**−1**
11	13	LKnee		−16
	34		RKnee	−2
12	6	RKnee		−7
	33		LKnee	2
13	33	RKnee		−2
	51		LKnee	**−1**
14	18	RKnee		3
	20		LKnee	2
15	13	RKnee		7
	17		LKnee	**1**
16	6	RKnee		24
	23		LKnee	−3
17	40	RKnee		−9
	83		LKnee	2
18	20	LKnee		**−1**
	75		RKnee	7
19	15	RKnee		−3
	75		LKnee	2
20	25	RKnee		−8
	43		LKnee	2
21	7	RKnee		2
	42		LKnee	2
22	20	RKnee		−8
	63		LKnee	−2

**Table 5:** Significance of pressure changes in stationary joint in different joint pairings

Pairing	Number of trials	Number of trials with pressure change ≥2 mmHg in stationary joints	Proportion of trials with pressure change in stationary joint
Ankle-Knee (ipsilateral)	14	12	0.86
Ankle-Hip (ipsilateral)	15	13	0.87
Knee-Elbow (ipsilateral)	15	12	0.8
RKnee-LKnee (contralateral)	22	17	0.77

**Table 6:** Dynamics of pressure change in driving and stationary RKnee-LKnee pair before and after transecting the periosteum

Animal#	Driving joint	Stationary Joint	Intact periosteum		Transected periosteum	
			t_r_	Δp	t_r_	Δp
7	RKnee		0:20	−8	0:18	−3
		LKnee	1:03	−2	5:22	**1**
8	RKnee		0:25	4	7:00	4
		LKnee	0:43	2	8:02	**−1**
9	RKnee		0:07	4	0:26	−5
		LKnee	0:42	2	7:42	**0**
10	RKnee		0:12	4	0:05	3
		LKnee	2:30	3	7:55	**1**
