# Supplementary material for: Subperiosteal Transmission Of Intra-Articular Pressure Between Articulated And Stationary Joints
Source: Sci Rep. 2015 Jan 29;5:8103. doi: 10.1038/srep08103 (PMC4648441; doi:10.1038/srep08103)
Supplement: Supplementary Information [file srep08103-s1.pdf]

SUBPERIOSTEAL TRANSMISSION OF INTRA-ARTICULAR PRESSURE BETWEEN ARTICULATED AND STATIONARY JOINTS

Mark Pitkin[1], Raghuveer Muppavarapu[2], Charles Cassidy[2], Emil Pitkin[3]

Department of Physical Medicine and Rehabilitation, Tufts University School of Medicine, Boston, MA, USA;
[2]Depatment of Orthopaedics, Tufts University School of Medicine, Boston, MA, USA;
[3]The Wharton School, University of Pennsylvania, Philadelphia, PA, USA.

Corresponding author: Mark Pitkin, 26 Mallard Dr., Sharon, MA 02067.
mpitkin@tuftsmedicalcenter.org

Legends for supplementary videos

SV1. Simultaneous measurements of pressure in the passively ranged right knee joint (RKnee) and in the stationary left knee joint (LKnee). Pressure changes in the right knee were from 0 to 4 mmHg; pressure changes in the left knee were from -1 to -3 mmHg. Venous blood pressure (right monitor) remained at the level of 37 mmHg.

SV2. Simultaneous measurements of pressure in the passively ranged right knee joint (RKnee) with cut periosteum above the joint and in the stationary left knee joint (LKnee). Pressure changes in the right knee were from 0 to -6 mmHg; pressure in the left knee remained unchanged at the level of -1 mmHg. Venous blood pressure (right monitor) was changing from 39 to 43 mmHg.
